# 1779. Trends in Anaplasmosis Over the Past Decade: A Review of Clinical Features, Laboratory Data and Outcomes

**DOI:** 10.1093/ofid/ofad500.1608

**Published:** 2023-11-27

**Authors:** Silpita Katragadda, Zachary A Yetmar, Supavit Chesdachai, Nischal Ranganath, Jonathan Wilcock, Omar M Abu Saleh, Douglas Challener

**Affiliations:** Mayo clinic, Rochester, Rochester, Minnesota; Mayo Clinic, Rochester, Minnesota; Mayo Clinic, Rochester, Minnesota; Mayo Clinic, Rochester, Minnesota; Mayo Clinic, Rochester, Minnesota; Mayo Clinic Rochester, Rochester, Minnesota; Mayo Clinic, Rochester, Minnesota

## Abstract

**Background:**

Human granulocytic anaplasmosis (HGA) is an emerging tick-borne illness endemic to the northeastern and midwestern United States. Risk factors for the severity of infection, predictors for hospitalization, temporal trends, co-infections, and outcomes are poorly studied.

**Methods:**

We conducted a retrospective review of HGA at Mayo Clinic, Rochester, between 2011-2021. Cases were identified by positive Anaplasma PCR at our reference laboratory. Variables included patients’ characteristics, symptoms, risk factors for tick-borne illness, laboratory parameters, co-infections, treatment, and clinical outcomes.

**Results:**

We identified 471 cases with a positive Anaplasma PCR, and 146 cases were included in this interim analysis. Table 1 shows a summary of the baseline characteristics. An increasing annual trend in diagnosis of HGA was noted from 2014 to 2016 with lower rates observed during COVID-19 pandemic (2020 to 2022) (Figure 1). Of the 146 patients, 19 (13%) were immunocompromised secondary to hematologic malignancies, solid organ malignancies, and pharmacological immunosuppression. Frequently reported symptoms included fever (79.5%), malaise (68.5%), chills (51.4%), and myalgia (50%). Tick exposure was documented in 79.2 % of cases. Forty-seven (33%) patients were hospitalized; older patients were more likely to be hospitalized (Table 2). The most common complications among the hospitalized patients included respiratory failure (50%), encephalopathy (44.4%) and renal failure (37.5%). One patient required ICU admission. Twenty-five patients (17.5%) and one patient (0.7%) were co-infected with Lyme and Ehrlichiosis, respectively. Higher rates of leukopenia and thrombocytopenia were associated with mono-infection with Anaplasma (p < 0.001). Follow up data was available for 136 patients; all of them had favorable response to doxycycline.

**Table 1:**
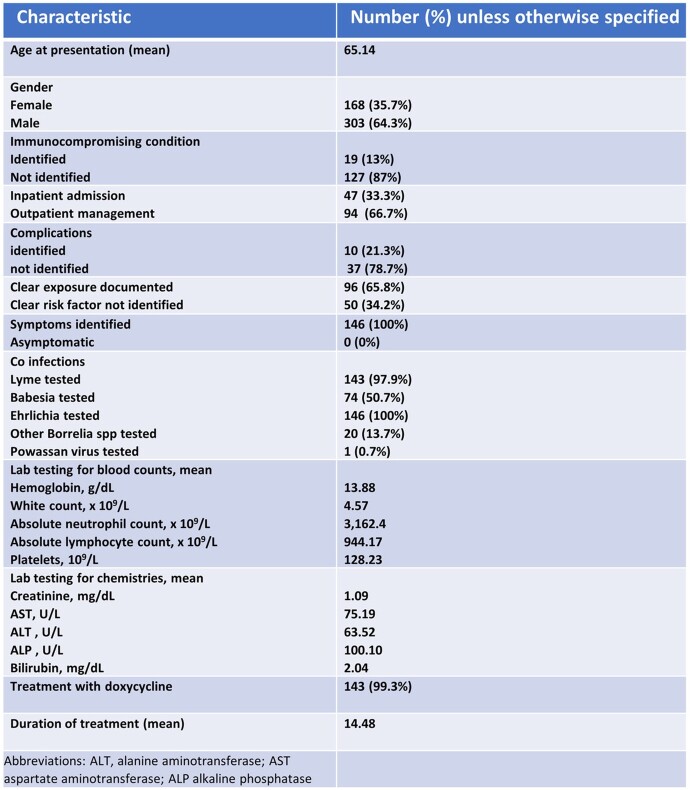
Summary of baseline characteristics, labs and treatment.

**Table 2:**
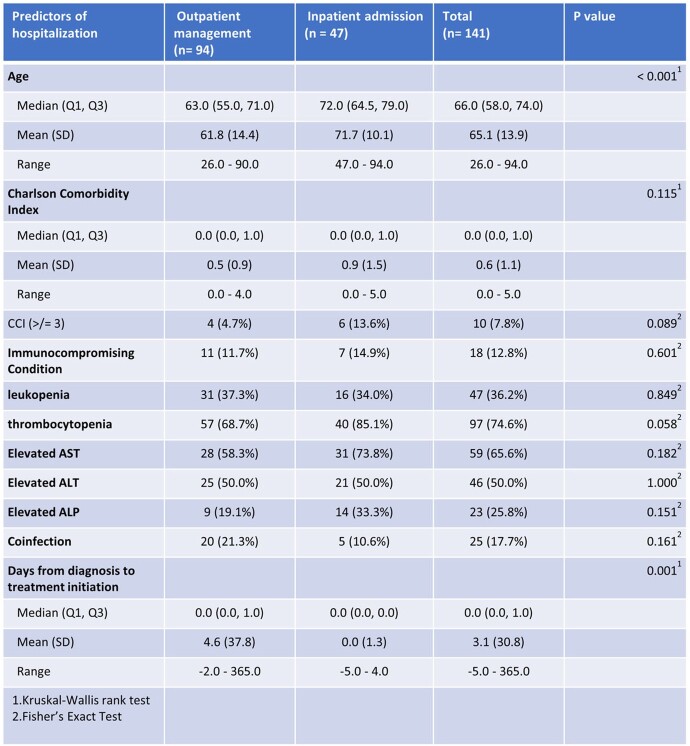
Predictors of Hospitalization

**Figure 1:**
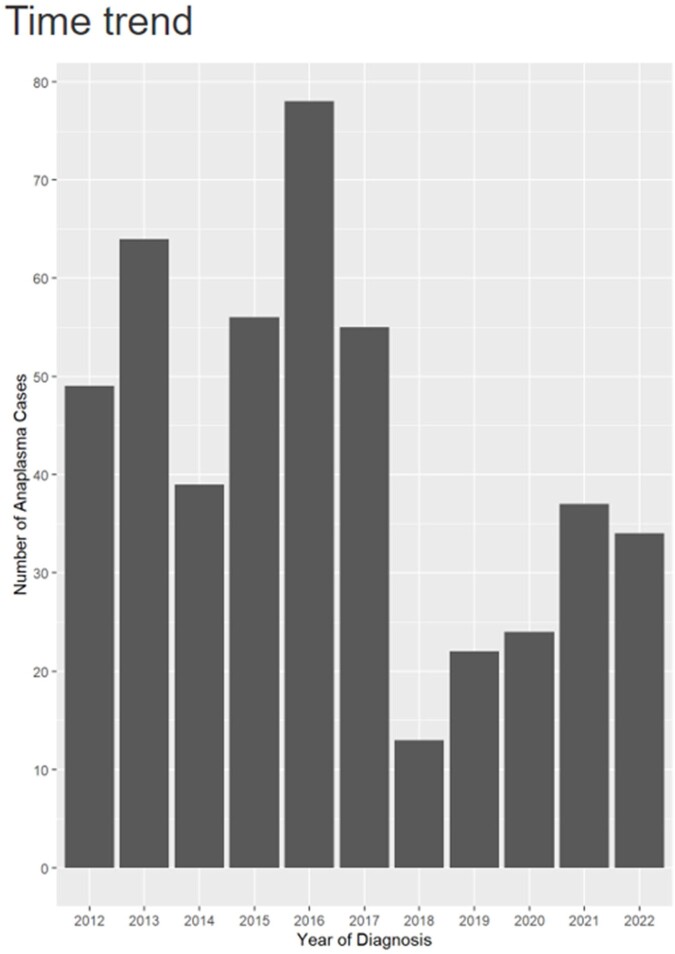
Annual Trends in Anaplasmosis from 2011-2021.

**Conclusion:**

Our study demonstrated comprehensive clinical characteristics of patients with HGA. Interestingly, tick exposure history was common, yet most patients did not have other tick-borne co-infections. Furthermore, only a minority of patients developed severe diseases and required hospitalization. The recent decline in cases during the early part of the COVID-19 pandemic is worth further investigation.

**Disclosures:**

**All Authors**: No reported disclosures

